# 53. Co-existence of systemic lupus erythematosus and primary sclerosing cholangitis in a 57 year old Maltese female

**DOI:** 10.1093/rap/rky034.016

**Published:** 2018-09-20

**Authors:** Mary Louise Camilleri, Zachary Gauci, Karen Cassar, Paul John Cassar

**Affiliations:** Rheumatology, Mater Dei Hospital, Malta, MALTA

**Introduction:** Systemic lupus erythematosus (SLE) and primary sclerosing cholangitis (PSC) are both uncommon conditions, with SLE having an estimated incidence of 1 to 10 per 100,000 person-years with female:male ratio of 8-15:1, whilst PSC occurs more rarely with an estimated incidence rate of 0.77 per 100,000 person-years and an incidence rate ratio for males versus females of 1.70 (1.34-2.07). Despite the rarity of the two conditions and the improbability of them coexisting in the same patient by chance, we report the coexistence of SLE and sclerosing cholangitis in a patient, who responded well to ursodeoxycholic acid.

**Case description:** We report the case of a 56 year old female patient with a history of SLE and anti-phospholipid syndrome since 18 years and 31 years of age respectively, who now presented with a one week history of worsening generalised abdominal pain. She had experienced similar episodes previously. Her past medical history included an episode of amaurosis fugax at age 31, right adrenal haemorrhage in 2011 that had rendered her dependent on steroids and arteritis of the left common and internal iliac arteries, which was confirmed on PET CT in 2015. She had also been investigated in view of a history of deranged liver function tests (LFT). At time of admission, she was taking prednisolone 10mg daily. Hydroxychloroquine had been tried unsuccessfully in the past but had been stopped due to severe intolerance, and she had been reluctant to start other disease-modifying agents to control her lupus activity. On presentation, she was haemodynamically stable and afebrile. Clinical examination was normal other than the presence of a malar rash, with her abdomen being soft and non-tender. She had a normal white count of 9, lymphocyte count of 1.04x10^9/L, haemoglobin of 9.7g/dL, MCV of 93fL and platelet count of 187 x10^9/L.. Erythrocyte sedimentation rate was moderately elevated at 56 mm/ 1stHr but the C-reactive protein was normal at 4.4mg/L. Liver function tests included a minimally raised bilirubin of 26.6umol/L, and alanine aminotransferase of 39U/L, whilst gamma-glutamyl transferase and alkaline phosphatase were moderately raised at 303U/L and 257U/L respectively. Renal profile and amylase were normal. A computed tomography (CT) scan of the abdomen demonstrated mildy dilated intrahepatic bileducts with a normal calibre common bile duct (6-7mm). There were no focal lesions in the liver and spleen. Small gallbladder stones were noted. The rest of the scan was normal. Further characterisation on magnetic resonance cholangiopancreaticography (MRCP) was consistent with a sclerosing cholangiopathy with multiple strictures, predominantly involving the right hepatic duct and its main branches, as well as the left hepatic duct and the segment II and III branches. Mild intrahepatic dilatation was noted secondary to the presence of the strictures, together with the presence of multiple cast calculi within these dilated ducts which were T1 hyperintense. There was no involvement of the extrahepatic biliary tree and the junction between the common bile duct and the pancreatic duct was normal. Gallstones were seen within the gallbladder with no signs of cholecystitis and no evidence of choledocholithiasis. There was no evidence of cirrhosis. The spleen was normal in size, and no focal liver lesions were identified. Comparison was made to the MRCP that had been performed two years earlier in December 2015 during investigation for deranged liver function tests and it was noted that whilst gallbladder stones were present, there had been no evidence of intrahepatic or extrahepatic biliary dilatation at this point. An oesophago-gastro-duodenoscopy at the time had not shown any pathology. Although a liver biopsy and colonoscopy had been offered to the patient, she had at the time declined, and also failed to attend for further investigation and follow up.In view of the new MRCP findings of sclerosing cholangitis, she was started on ursodeoxycholic acid (UDCA) 750mg daily, with marked improvement in the liver function tests as shown in the table below. Azathioprine was subsequently introduced in March 2018 as a steroid sparing agent, but this had to be stopped as shortly after its introduction the patient developed lethargy, nausea and severe bruising which settled on cessation of the azathioprine.

**Discussion:** The liver may be affected by a wide variety of rheumatic diseases, and dysfunction may be due to the toxic effects of pharmacotherapy or related to the autoimmune disease. Liver dysfunction may also occur as a primary disorder in association with rheumatic disease. Abnormality of hepatic function in association with SLE has been described as early as 1955 and is reported in as many as 25-50% of patients with lupus. In most cases, this is only subclinical and end stage liver disease is rare in SLE. The liver-related complications in SLE includes lupus hepatitis as well as autoimmune liver diseases such as autoimmune hepatitis, primary biliary cirrhosis and primary sclerosing cholangitis (PSC). Steatohepatitis, fatty liver and drug-induced liver injury may also occur. Distinguishing between them is often challenging. To date, few literature reviews and case reports of concomitant PSC and SLE have been published. Although a liver biopsy is diagnostic of PSC by the presence of typical onion-skinning appearance and periductal inflammation, diagnosis is nowadays established with non-invasive imaging study, typically, an MRCP as was done in this patient. Possible associations between PSC and SLE have been noted in the literature. The most common genetic findings in primary sclerosing cholangitis have been identified within the HLA complex on chromosome 6 with an odds ration of 3-5. However, in a comprehensive review article, it was demonstrated how candidate genes outside of this complex have been reported in both PSC and a number of auto-immune diseases based on mapping of genome-wide significant risk loci. Twenty three genome-wide significant (p ≤ 10-8) risk loci outside of the HLA complex on chromosome 6 have been reported together with the associated candidate genes, and the P value for the single nucleotide polymorphism within this implicated risk region in PSC. Of relevance to this case, an association has been made between the candidate genes for both PBC and SLE. These genes include SH2B3 and ATXN2 on chromosome 12q24 and CLEC16A and SOCS1 on chromosome 16q12. Thus, this might prove that there is common genetic susceptibility towards developing both SLE and PSC.

**Key Learning Points:** It is still unclear whether PSC occurring in relation to SLE is coincidental or if there is a casual association between the two conditions. More research in this field may yield more clues about the possible genetic link between these two diseases.

**Disclosure: M. Camilleri:** None. **Z. Gauci:** None. **K. Cassar:** None. **P. Cassar:** None.

**Table 1:** Trend of liver enzymes over time

	Range	2/11/2017	15/1/2018	18/1/2018	10/2/2018	11/3/2018	06/4/2018	12/5/2018	26/5/2018
Bilirubin (Umol/l)	1.0-17	32.8	23.5	26.1	28.8	29.7	34.5	29.8	31.7
ALP (U/l)	40-129	251	257	189	108	122	142	163	149
GGT (U/l)	11.0-50	525	303	272	87	111	97	115	100
ALT (U/l)	1.0-41	50	39	30	10	15	16	15	14

